# Social and Cultural Elements Associated with Neurocognitive Dysfunctions in Spinocerebellar Ataxia Type 2 Patients

**DOI:** 10.3389/fpsyt.2015.00090

**Published:** 2015-06-10

**Authors:** Roberto Emmanuele Mercadillo, Víctor Galvez, Rosalinda Díaz, Lorena Paredes, Javier Velázquez-Moctezuma, Carlos R. Hernandez-Castillo, Juan Fernandez-Ruiz

**Affiliations:** ^1^Laboratorio de Neuropsicología, Departamento de Fisiología, Facultad de Medicina, Universidad Nacional Autónoma de México, Mexico City, Mexico; ^2^Consejo Nacional de Ciencia y Tecnología-Cátedras, Mexico City, Mexico; ^3^Área de Neurociencias, Departamento de Biología de la Reproducción, Universidad Autónoma Metropolitana, Unidad Iztapalapa, Mexico City, Mexico; ^4^Posgrado en Neuroetología, Universidad Veracruzana, Xalapa, Mexico; ^5^Facultad de Psicología, Universidad Nacional Autónoma de México, Mexico City, Mexico; ^6^Instituto de Neuroetología, Universidad Veracruzana, Xalapa, Mexico; ^7^Facultad de Psicología, Universidad Veracruzana, Xalapa, Mexico

**Keywords:** spinocerebellar ataxia type 2, neurodegeneration, ethnography, culture, social neuroscience

## Abstract

Spinocerebellar Ataxia Type 2 (SCA2) is a rare genetic disorder producing cerebellar degeneration and affecting motor abilities. Neuroimaging studies also show neurodegeneration in subcortical and cortical regions related to emotional and social processes. From social neuroscience, it is suggested that motor and social abilities can be influenced by particular cultural dynamics so, culture is fundamental to understand the effect of brain-related alterations. Here, we present the first analysis about the cultural elements related to the SCA2 disorder in 15 patients previously evaluated with neuroimaging and psychometric instruments, and their nuclear relationships distributed in six geographical and cultural regions in Mexico. Ethnographic records and photographic and video archives about the quotidian participant’s routine were obtained from the patients, their relatives and their caregivers. The information was categorized and interpreted taking into consideration cultural issues and patients’ medical files. Our analyses suggest that most of the participants do not understand the nature of the disease and this misunderstanding favors magic and non-medical explanations. Patients’ testimonies suggest a decrease in pain perception as well as motor alterations that may be related to interoceptive dysfunctions. Relatives’ testimonies indicate patients’ lack of social and emotional interests that may be related to frontal, temporal, and cerebellar degeneration. In general, participants use their religious beliefs to deal with the disease and only a few of them trust the health system. Patients and their families are either openly rejected and ignored, tolerated or even helped by their community accordingly to different regional traits. We propose that ethnography can provide social representations to understand the patients’ alterations, to formulate neurobiological hypotheses, to develop neurocognitive interventions, and to improve the medical approach to the disease.

## Introduction

Spinocerebellar Ataxia Type 2 (SCA2) is a rare neurodegenerative disease with a high incidence in Mexico (1). Its symptoms regularly start during the third or fourth decade and include several motor and visuomotor deficits, such as ataxia, dysmetria, dysarthria, dysdiadochokinesia, and prolonged latency of saccadic ocular movements (2–5). The genetic cause of SCA2 involves mutations in the CAG trinucleotide repetitions in gene encoding ataxin-2 (6, 7) primarily causing cerebellar degeneration in the Purkinje cells and the brainstem (8, 9).

During the past decade, structural neuroimaging techniques have been used to identify loss of gray matter in different SCA types (10–12). Regarding SCA2 patients, some studies report gray matter loss in the pons and the cerebellar vermis compared with healthy volunteers (13, 14). When comparing different SCA types using Voxel Based Morphomety (VBM) patients diagnosed with SCA1, SCA2, and SCA3 present atrophy of the cerebellum and brainstem, and SCA2 patients reveal the largest atrophy (15). In addition, brain deterioration in SCA2 patients is not restricted to the cerebellum and brainstem but includes cortical regions such as, orbitofrontal, middle frontal, primary sensorimotor, temporomesial, and insular cortices (14, 16).

Neural deterioration inferred with VBM can be used to complement the discussion on the behavioral and cognitive dysfunctions in SCA2. This is important since conditions resulting from cerebellar and non-cerebellar damage may occur in parallel in this disease (17). For example, alterations in motor and learning processes can also be regulated by extra-cerebellar structures, such as the substantia nigra, striatum, pallidum, and motor cortex (18). In addition, atrophies in the posterior and anterior cerebellar lobes have been related to executive and coordinative dysfunctions in SCA2 patients (19). Also, patterns of cerebellar and non-cerebellar morphological alterations are not only associated with visual motor incoordination but also with non-motor functions such as, verbal and visual memory, attention, learning abilities, and language comprehension (20–22). Furthermore, several impairments in cognitive social abilities, such as, Theory of Mind and difficulties in recognizing the social emotional expressions of others have also been observed in SCA patients (23–25).

Results derived from neuroimaging studies can also be useful to discuss emotional disturbances in patients with SCA2 and other cerebellar alterations. For example, cerebellar lesions influence affective recognition (26, 27) and the vermis has been proposed as the limbic cerebellum to regulate emotional expressions (28). Observations in neurological patients with morphological and functional alterations in the vermis and diagnosed with ataxia or with cerebellar cognitive affective syndrome, manifest emotional fragility and several affective disorders (29–32). Furthermore, some functional neuroimaging studies indicate cerebellar activity when individuals watch pictures of faces with emotional content (33) and while feeling anger, sadness, happiness, and fear (34, 35). In social cognition, the role of the cerebellum has been observed along with the activation of the hippocampus while processing socially related spaces (36) and along with activity of the prefrontal cortex predicts autonomic responses associated with risky social decision-making (37).

Some findings suggest that SCA2 affects not only the motor and cognitive domains, but could also have a profound impact in social expressions as well. Previous studies have explored the correlation between clinical and socio-demographic features with cognitive social impairment (23, 24). However, their interpretations do not take into account the patient’s social environment, and are mainly based on social cognition abilities tests.

A more comprehensive theoretical schema to assess the relationship between social cognitive elements and brain deterioration in SCA2, may be a social neuroscience approach. This approach considers that social dynamics contextualize, motivate, and influence cognition, emotion, and brain functions, which may result in a variety of inter-subjective interactions manifested in social behavior (38). From this perspective, the study of social cognition and behavior must be comparative since similar circumstances can elicit diverse responses between different individuals or groups (39, 40). Consequently, different social environments may influence cognitive deterioration or manifestations evaluated in SCA2, as it has been previously proposed for other neuropsychiatric disorders (41).

Social cognition involves several processes and brain regions that are affected in SCA2. Among them individuals’ own and others’ motor representations regulated by the mirror neuron system, introspective processes controlled by sensoriomotor, prefrontal, cingulate, and insular cortices (42, 43). Also include homeostatic and emotional reactions, and top-down effects of controlled processes involving intentions, decision-making, affective evaluations, memory, and social values (44). The complexity of these processes involves the interplay of both biologically evolved neurocognitive systems and cultural mechanisms. Neurocognitive systems can include learning, memory, or perceptual systems selected to process specific physical and social information (45). Cultural mechanisms involve patterns of beliefs, emotions, practices, speeches, and interpersonal relations, which influence cognition and organize behavior according to the particular history of individuals and groups (46, 47).

Therefore, although social neuroscience mainly concerns brain anatomy and function, the field requires collecting extra-cerebral, behavioral, and social data. This entails interdisciplinary work since, as proposed from anthropological perspectives, the individual’s experience does not depend solely on brain functions, even though these are obligatory for any cognitive and behavioral manifestation. In this perspective, the individual is considered as a self, or an entire organism including the biological, psychological, social, and cultural elements needed to integrate awareness and allow autobiographical narrations and ethical values to be questioned and defined (48). From this point of view, social cognition may represent elements grouped in a gestalt functioning as a mnemonic code into a structure, which establish several nets reflected in collectivity, organization, multiplicity, and variability (49). These anthropological cognitive premises are relevant to the study of SCA2 since patients’ explanations about the disease and suffering are made from symbolic descriptions that model their experiences (50). In this sense, the notion of a health social system considers the institutions’ and professionals’ role in dynamics favoring health. The notion of a health cultural system emphasizes the symbolic dimension of people’s perceptions and knowledge around the concept of health and disease (51).

An approach to study and recognize the individual’s social representations is an ethnographic method. This implies a related anthropological view by recording first-person knowledge and testimonies to interpret life experiences, values, and worldviews of people in a given cultural and ecological environment (52). Ethnography agrees with the recent neuroanthropological proposal that argues that culture involves psychological mechanisms when transmitting and sharing information between people who possess specific biological requirements (53, 54). By using ethnography, brain function could be interpreted as an expression of particular variables of social and cultural life and would also have an impact on biomedical and neurological research since health is not only considered as biological but as a biopsychosocial state. Research on health elaborated from this point of view aims to understand a disease and improve treatments. Also, include the patients’ view and needs in this improvement which may be accessible only through the experiences of patients living with the disease, particularly important in cases of degenerative ataxias (55). This view may also lead to an understanding of the organizational behavior of the patients and people around them in order to identify and eventually modify their attitudes toward the disease (56).

Ethnography is based on qualitative records and systemic-wide analysis of people and cultures. Unlike quantitative methods used in biomedical research, this qualitative approach observes subtle particularities of the cultural phenomena, creating a detailed representation of the portrait of a specific group (57, 58).

In this work, we present an integrative analysis to elucidate if ethnographical methods can complement neuroimaging and psychometric evaluations to understand neuropsychiatric diseases, particularly SCA2. To do this, we describe and interpret personal, social, and cultural attributions associated with SCA2 in patients and people surrounding their nuclear relations using the aforementioned interdisciplinary methodology. Our descriptions and interpretations are related with the neurodegeneration identified by VBM in these patients. Discussions on the impact of biomedical research in the patient’s daily life are elaborated.

## Materials and Methods

### Participants

We invited 15 patients from 6 different Mexican geographic regions to participate in this study, which is a part of a larger investigation on the neurocognitive deterioration associated with several SCA types (socio-demographic data and details about the patients are presented in Tables 1–6). Patients (nine women; 37.2 ± 15.9, max. 65, min. 15 years old) had a molecular diagnosis of Spinocerebellar Ataxia Type 2 indicating the expanded repetitions of the CAG trinucleotide in the gene ATXN2 (locus 12q24) (max. 48, min. 37 CAG repetitions) (CAG repetitions for each patient are indicated in Tables 1–6). Patients were contacted and recruited by members of the research team based on the database of National Institute of Neurology and Neurosurgery (NINN). A number of patients who were not part of the NINN database were contacted through social and institutional networks established during the study. The research protocol was conducted according the international directions in the Declaration of Helsinki and procedures were approved by the Health and Ethics Committees of the National Autonomous University of Mexico.

**Table 1:** **General description of the participants living in Mexico City, neighborhood Copilco**.

I.D.	SCA2 diagnosis	Disease evolution (months)	Gender	Age (years)	Connection	Education (years)	Occupation	Remarks
P01	Yes (51 CAG repetitions)	60	Female	29	Focal patient	9	Housewife	She arrived at the National Institute of Neurology and Neurosurgery (NINN) in 2008 to be genetically diagnosed with SCA2 due to advanced symptoms that resembled her mother
P02	Yes (52 CAG repetitions)	60	Male	20	Brother	6	–	He arrived at the NINN in 2008 following his sister’s example, but he refused to be evaluated in depth and to receive treatment until the members of the research project asked him to participate
P03	No	–	Male	58	Father	6	Bricklayer and cook	He is both patients’ caregiver

General characteristics: The nuclear family lives together in the same home and is made up of the three people described above and the patients’ mother with a SCA2 diagnosis and with a complete inability to walk and talk. The non-symptomatic patients’ sister (32-year-old) helps their father care for her siblings occasionally and is a source of financial support since most of the father’s time is used to care for the patients. Low socioeconomic and educational level is reported in the family’s institutional file. The patients’ mother receives medical check-ups and support at home. No other SCA2 precedent is mentioned.

*Location general description: Copilco el Alto is one of Mexico’s oldest neighborhoods located in the Coyoacán borough in Mexico City. It is mostly a low socioeconomic level neighborhood with approximately 1684 inhabitants. About 15 min are needed to arrive to the NINN*.

**Table 2:** **General description of the participants living in Mexico City, neighborhood Del Valle**.

I.D.	SCA2 diagnosis	Disease evolution (months)	Gender	Age (years)	Connection	Education (years)	Occupation	Remarks
P04	Yes (40 CAG repetitions)	252	Female	65	Focal patient	8	–	She arrived at the National Institute of Neurology and Neurosurgery in 1995. Her file is the most complete among the participants, it includes monthly revisions, and several anxiety and depressive episodes associated with insomnia are mentioned
P05	No	–	Male	44	Son	16	Creative in cinematographic industry	He has not manifested any symptoms and qualified as negative according to the SCA2 genetic test
P06	No	–	Female	45	Caregiver	12	Caregiver	She has been the patient’s daily caregiver for the past 6 years

*Family general characteristics: The focal patient is a widow who has two sons ages 44 (P05) and 43. Neither son manifests SCA2 symptoms and both qualified as negative according to the SCA2 genetic test. The patient used to go to the NINN to be constantly evaluated and to be with other patients and people whom she perceives as tolerant. She cannot walk and manifests severe difficulties when speaking and coordinating movements. She lives at home with her caregiver and is constantly visited by her sons and grandsons. The family qualifies as being middle class. No other SCA2 precedent is mentioned. Location general description: Del Valle is a neighborhood located in the Benito Juarez borough in Mexico City. People living there are usually considered as have a mid-socioeconomic and educational level. It has an area of 16 km^2^ with 46,900 inhabitants. About 25 min is needed to arrive at the NINN*.

**Table 3:** **General description of the participants living in La Moncada**.

I.D.	SCA2 diagnosis	Disease evolution (months)	Gender	Age (years)	Connection	Education (years)	Occupation	Remarks
P07	Yes (42 CAG repetitions)	120	Female	46	Focal patient	6	Housewife	She arrived at the National Institute of Neurology and Neurosurgery in 2005 with alterations of language, hyporeflexia, and a diabetes diagnosis. At that moment she began regular annual medical check-ups. In 2011 she began physical therapy in her community medical center
P08	Yes (43 CAG repetitions)	2	Male	19	Son	9	Farmer and goat breeder	He was diagnosed with SCA2 in 2014 when the project’s members invited him to participate in the research
P09	Yes (41 CAG repetitions)	156	Female	44	Sister	9	Housewife	She is a widow first diagnosed as a drug abuser at 30-year-old when she was living in the U.S. with her three children (patient P10 and two non-symptomatic 9- and 11-year-old boys). She was officially diagnosed with SCA2 in 2014 when the project’s members invited her to participate in the research
P10	Yes (46 CAG repetitions)	24	Female	15	Niece (P09’s daughter)	9	Student	She was diagnosed with SCA2 in 2013 when her family carried her to the NINN due to instability while walking. She receives physical rehabilitation in her community medical center
P11	Yes (39 CAG repetitions)	24	Female	48	Sister	8	Housewife	She is a widow living in Dallas, TX, USA, but she came to La Moncada to be diagnosed with SCA2 in 2014
P12	No	–	Male	47	Brother	9	Municipal policeman	He is a non-symptomatic family member who mainly cares for and economically supports his sister P09
P13	No	–	Female	36	Sister	9	Housewife and merchant	She is a non-symptomatic family member who takes care of her sisters P09 and MG and helps them in their household activities

*Family general characteristics: The complete nuclear family consists of eight siblings of whom P09, P07 and P15 present SCA2 symptoms. P13 and P12 live in La Moncada and care for their sisters while the other three siblings live in the U.S. Several members of the extended family (nieces and nephews) live in the U.S. but, as the participants indicated, they are returning to La Moncada since they suspect they have SCA2. The focal patient´s father and grandfather died manifesting symptoms that resembled SCA2. Patient P09 lives with her three sons, patient P07 lives with her son and her husband. P13 and P12 live near their sisters. Location general description: La Moncada is a little village with 4130 inhabitants belonging to the Municipality of Tarimoro in the State of Guanajuato, with 35,571 in 2010 according to the Social Development Ministry. Fifty-eight percent of the population is classified as poor, the educational level is 6.2 years of study, and 55 medical personnel assist the population. The main economic activities in the municipality include animal breeding and agriculture. Five hours is the approximately time when traveling by car from La Moncada to the NINN*.

**Table 4:** **General description of the participants living in Tlaltetela**.

I.D.	SCA2 diagnosis	Disease evolution (months)	Gender	Age (years)	Connection	Education (years)	Occupation	Remarks
P14	Yes (37 CAG repetitions)	528	Male	58	Focal patient	1	Farmer	He was diagnosed with SCA2 in 2012. He has been examined by the regional medical service four times but he has not received treatment
P15	Yes (42 CAG repetitions)	96	Female	33	Daughter	3	Housewife	She was diagnosed in 2012 when the project’s members invited her to participate. She has not received treatment. She is a single mother with a 12-year-old child
P16	Yes (45 CAG repetitions)	84	Male	21	Son	3	Farmer	He was diagnosed in 2012 when the project’s members invited him to participate. He has not received treatment
P17	Yes (42 CAG repetitions)	48	Male	29	Son	3	Farmer and bricklayer	He was diagnosed in 2012 when the project’s members invited him to participate. He has not received treatment

*Family general characteristics: The whole family is made up of nine members: father (focal patient), mother and seven siblings. Patient P14 lives in the same home with his wife, his sons P15 and P16, and his grandson (P15’s son). They live in La Represa which is a group of little ranches 15 min from Tlaltetela, the nearest town. P17 lives in Tlaltetela with his wife and eight young children. The rest of the family lives in other cities in the north of the country. The focal patient indicated that his father and five uncles died with symptoms that resembled SCA2. Location general description: La Represa has 75 inhabitants near the Municipal Head called Tlaltetela where 55 SCA7 and 9 SCA2 patients were reported by Magaña et al. (59). Tlaltetela is a municipality with 4528 inhabitants in 2010 and belongs to the State of Veracruz of Ignacio de la Llave. 19.6% of the population has 6 years of education, 86% are classified as poor and there are 0.5 medical personnel per 1000 inhabitants distributed in three medical public units. The main economic activities include sugar cane and corn agriculture, and swine breeding. Seven hours is the approximate time needed to travel by car from Tlaltetela to the NINN*.

**Table 5:** **General description of the participants living in San Nicolas Coatepec**.

I.D.	SCA2 diagnosis	Disease evolution (months)	Gender	Age (years)	Connection	Education (yeas)	Occupation	Remarks
P18	Yes (39 CAG repetitions)	216	Female	60	Main patient	2	Housewife	She arrived at the National Institute of Neurology and Neurosurgery in 2012 and was diagnosed with SCA2. Relevant aspects of her institutional file indicate speech alterations, forgetfulness and dyspnea. She denies alterations in vision, hearing and sensitivity
P19	Yes (45 CAG repetitions)	96	Male	35	Brother	6	Food merchant	He arrived at the NINN in 2013 and was diagnosed with SCA2. The relevant aspect in his institutional file indicates he abused alcohol, inhalants and marihuana during his adolescence. He smokes one cigarette packet per day
P20	No	156	Male	41	Brother	6	Gardener	He does not want to be officially diagnosed but he manifests evident motor and speech alterations resembling SCA2
P21	No	–	Female	33	Sister in law	6	Food merchant	She has been P19’s wife for 13 years and is her husband’s caregiver
P22	No	–	Male	57	Husband	2	Bricklayer	He has been married 36 years with P18
P23	No	–	Female	27	Daughter	9	Housewife	She has a young child and is her mother’s main caregiver
P24	No	–	Female	21	Daughter	9	Housewife	

*Family general characteristics: Three nuclear families integrate the participant’s extended family. P18’s nuclear family includes P22, P23, P23’s son, P24 and two brothers not included in the interview and who work as bricklayers and live in the same home; P19’s family is made up of his wife and their four young children; P20’s family is his wife. The three families make up an extended family who lives close to each other and cooperate. In general terms they have good relationships and low socioeconomic level. Location general description: San Nicolas Coatepec is a town with 4007 inhabitants located in the Tianguistenco Municipality belonging to the State of Mexico. Two hundred and fourteen inhabitants are illiterate. The main economic activities are agriculture and commerce and it is a Mexican municipality that has a high percentage of migration to the U.S. and immigrants’ incomes represent an important source of financial resources. Two hours is the approximate time needed to travel by car from Coatepec to the NINN*.

**Table 6:** **General description of the participants living in Ezequiel Montes**.

I.D.	SCA2 diagnosis	Disease evolution (months)	Gender	Age (years)	Connection	Education	Occupation	Remarks
P25	Yes (48 CAG repetitions)	276	Female	41	Focal patient	6	She used to help sell candies at home 4 years ago but not anymore	She arrived at the National Institute of Neurology and Neurosurgery in 2002 and was diagnosed with SCA2 in 2009
								She and her family never returned to the institute and no medical attention was given during those years
P26	No	–	Female	76	Mother	3	Housewife	She is the main patient’s caregiver and has diabetes
P27	No	–	Female	39	Sister	9	Merchant	She does not have SCA2 following a medical test. She has a 9-year-old child
P28	No	–	Female	40	Sister	9	Merchant	She does not have SCA2 following a medical test. She has two 16- and 15-year-old girls

General observations: Participants indicated that the disease initiated with the focal patient’s father’s great-grandmother. Some members of the nuclear family have presumably died because of the disease: Father (it began at 30-year-old and he died at 50-year-old), the eldest sister (it began at 18-year-old and she died at 23-year-old), brother (it began at 10-year-old and he died at 25-year-old) and the youngest sister (it began at 2-year-old and she died at 10-year-old). The family indicated that they are the only ones who have the disease in the town but that many of the patient’s father’s relatives present with the disease in Villa Progreso, a small group of villages near Ezequiel Montes.

*Location general description: Ezequiel Montes is the head of the municipality of Ezequiel Montes with 13,900 inhabitants in the State of Querétaro. The main economic activities include bovine farming, grapevine agriculture and a small exploitation of lime, opal, and sand for construction. Recently some industries of cloth, plastic, and food for livestock have been established. Four hours is the approximate time needed to travel by car from Ezequiel Montes to the NINN*.

### Psychometric and brain structural participant’s characteristics

**Figure 1:**
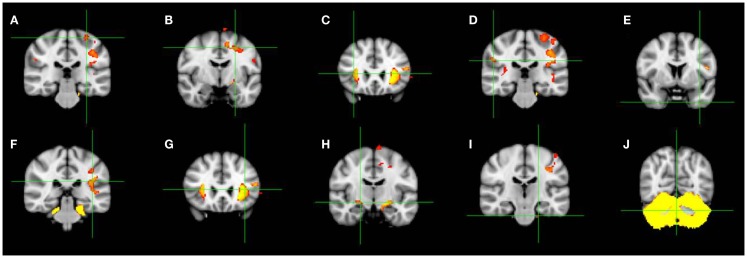
**Coronal slices showing brain regions with significant gray matter volume reductions in the 15 SCA2 patients participating in this study as observed by using VBM**. Green crosshairs indicate the peak differences. **(A)** Left precentral gyrus, **(B)** left middle frontal gyrus, **(C)** right inferior frontal gyrus, **(D)** right inferior parietal gyrus, **(E)** middle temporal gyrus, **(F)** right insula, **(G)** left insula, **(H)** right parahippocampal gyrus, **(I)** left brainstem (pons), **(J)** left posterior cerebellum (vermis) [modified from Mercadillo et al. (60)].

All patients participating in this social study were previously evaluated by our group using psychometric, motor, and cognitive tests. Also, brain degeneration in these patients was identified using the VBM technique. Details of the behavioral, cognitive, and brain degeneration characteristics can be observed in Mercadillo et al. (60). Below, we resume the results of these evaluations and an illustration of the main brain degenerated regions is presented in Figure 1 (more information in Supplementary Material).

All patients were evaluated using the Scale for Assessing and Rating Ataxia (SARA) (61, 62), showing the motor alterations classically associated with SCA2 (mean score: 17.2 ± 8.5; max. 33.5, min. 2). The 15 patients and 15 control-matched volunteers also responded to the Mini-mental State Examination (MMSE) validated in Mexico to assess cognitive impairment (63), the Montreal Cognitive Assessment (MoCA) to complement the MMSE evaluation (64), and The Cambridge Neuropsychological Test Automated Battery (CANTAB) consisting of a computerized touch system to assess cognitive changes (65). For our study, we considered three CANTAB tasks: Big/Little Circle evaluating comprehension and learning while training the participant to follow and reverse a rule; the Spatial Span and the Intra/Extra Dimensional Shift which is analog to the Wisconsin Card Sorting test to assess visual discrimination, ability to maintain attention, shifting, and flexibility of attention.

General psychometric results showed that SCA2 patients exhibited lower scores than control participants in the MMSE (control: *M* = 29.06 ± 2.05; patients: *M* = 25.80 ± 3.42; *t*_28_ = 3.16, *p* = 0.004) and in the MoCA (control: *M* = 28.06 ± 1.62; patients: *M* = 21.06 ± 6.12; *t*_28_ = 4.27, *p* < 0.001) indicating cognitive impairments. Also, patients made more errors and required more time to respond in the CANTAB tests. These results support previous findings from our group and others in which SCA2 patients exhibit low performance in non-verbal abilities (66). In addition, these cognitive abilities get worse as the motor difficulties and disease advance (67).

All the patients and matched volunteers were scanned using a 3 Tesla Philips Achieva MRI instrument (Philips Medical Systems, Eindhoven, The Netherlands) to compare and analyze gray matter reductions using the VBM technique (68). As previously reported, the VBM analysis revealed significant degeneration in the cerebellar vermis and brainstem, and in cortical and subcortical regions involving insula and parahippocampal gyrus, as well as precentral, frontal, parietal, and temporal cortices (see Figure 1).

### Qualitative and ethnographic inquiry

The ethnographic qualitative research was based on The Grounded Theory focused on elaborating notions about the individual’s social representations based on symbolic interactionism. This theory proposes that people act over the situations affecting them according to their own understandings and the meanings of the elements that make up the environment around them. These understandings and meanings emerge from social interactions observed and recorded by the research to be used and transformed through interpretative processes that people use to face specific situations (50, 69).

**Figure 2:**
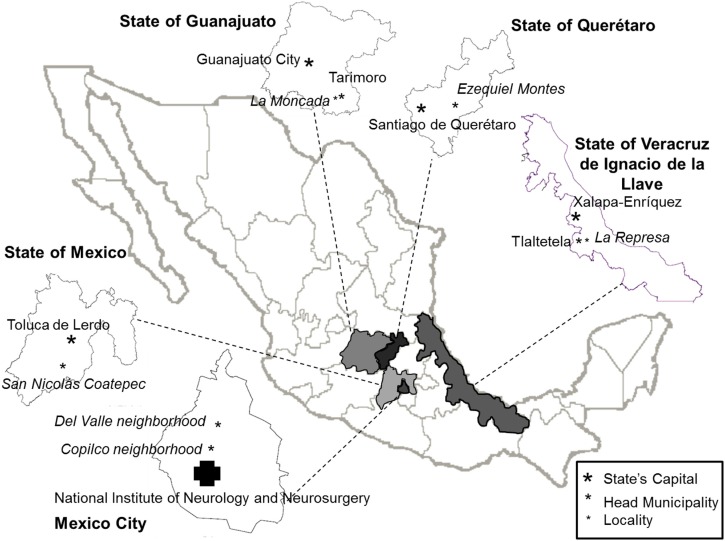
**Map of the Mexican Republic illustrating the six geographical regions where the 15 patients and their relatives participating in the study live**.

Field observations were carried out in 2013 and 2014 in the environments surrounding the patients who agreed to interact with researchers and expressed interest in the research. We also invited 13 relatives and caregivers that make up the patients’ main and nuclear interpersonal relationships. Descriptions about the patients, relatives, and caregivers, as well as general descriptions of their social and geographical context are presented in Figure 2 and Tables 1–6.

We conducted individual interviews when possible or group interviews when the patients needed help answering questions due to speech deficiencies. The group dynamics allowed dialog and discussion among participants about differences on perception, emotional experience, attitudes, and related decisions. All the interviews were audio-recorded for posterior analysis of content. The interviews involved general questions that emerged from the researchers’ interest in social cognition and neurocognitive impairments in SCA2, and the concern transmitted by clinicians and some patients and relatives about their necessities and doubts communicated to the clinicians. Thus semi-structured interviews were performed and audio-recorded with a previously designed script focusing on the following issues: 1. Family structure, 2. Concept and definition of the disease; 3. Evolution of the disease; 4. Consequences of having the disease; 5. Ways of facing the consequences; 6. Known people with similar disease manifestations; 7. Perception of the public health services; 8. Future prospects.

Participant observation (70) was also conducted during the field work at the participants’ home and quotidian spaces, as well as, during clinic, psychometric, and neurological evaluations. Observations were recorded in a field diary and photographic and video recordings were also collected for posterior analysis.

In all cases, the participants’ testimonies were confidential and they were informed that the material would only be used as part of this investigation without any negative consequence for their medical care.

Behavioral records and interviews were read and analyzed separately by two members of the team with experience in qualitative analysis (Roberto Emmanuele Mercadillo and Lorena Paredes) in order to identify the main variables that affected the general questions. New interviews and field observations were performed when needed. The subsequent observations and records were made according to the diversity of variables identified in the previous analyses and ended when these variables were observed as analogous. The variables obtained in the analyses were divided in categories that reflected the main issues perceived by the participants.

To complement this field research, the files describing the patient’s clinic history in the NINN were reviewed.

## Results

The ethnographic inquiry regarding the patient’s and their relatives’ perceptions involves inseparable relational processes. The results of our inquiry are presented in the categories below that originated from the analysis of the interviews and field observations concerning: the participant’s perceptions and understandings about the concept of spinocerebellar ataxia, the evolution of the disease, their feelings, and their social and institutional perceived support. Results are presented in a typical ethnographic narrative and some quotes and translations of the participants’ testimonials are shown to illustrate our interpretations.

### The concept and the evolution of the disease

**Figure 3:**
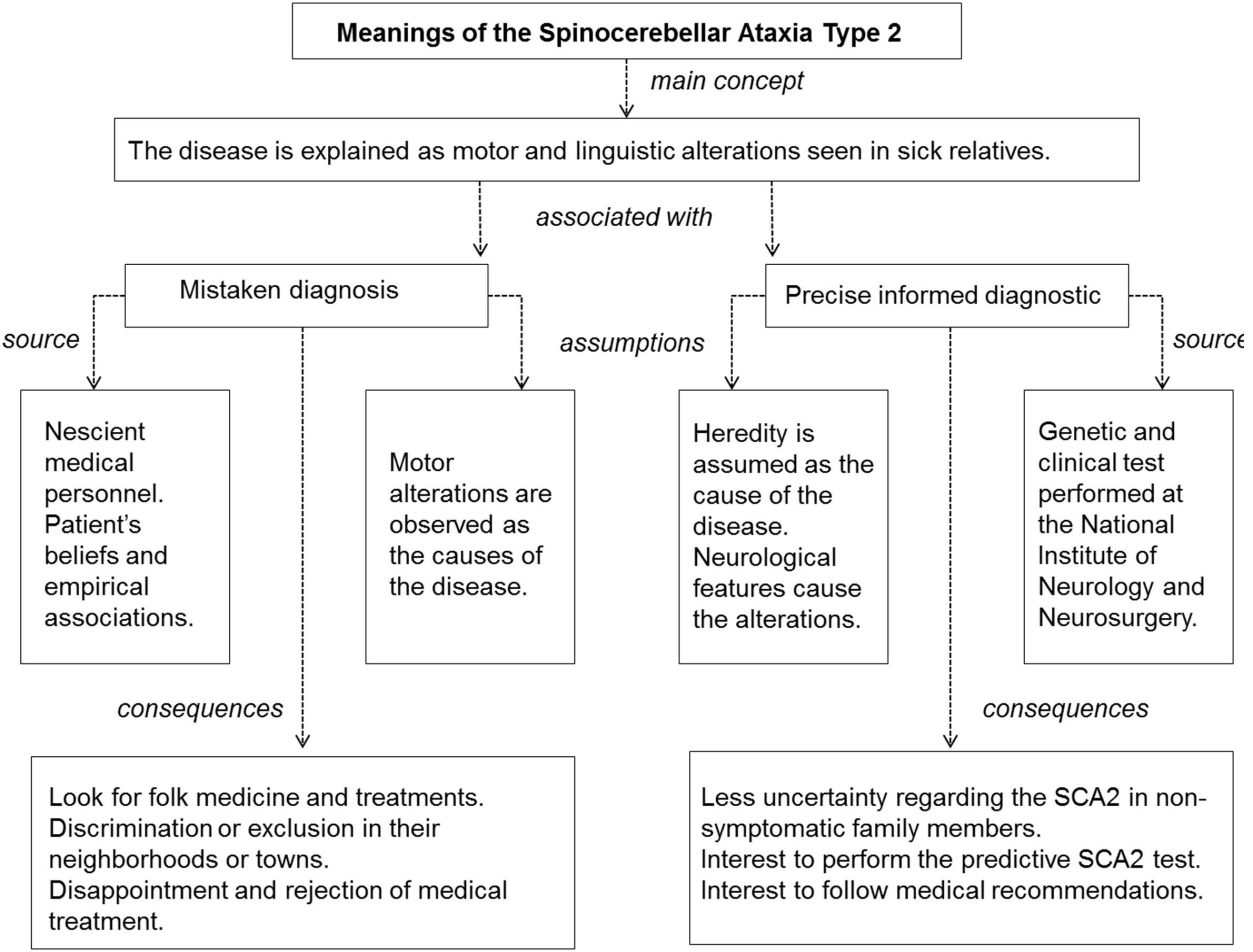
**Summary of the main participants’ meanings and concepts on spinocerebellar ataxia Type 2**.

**Figure 4:**
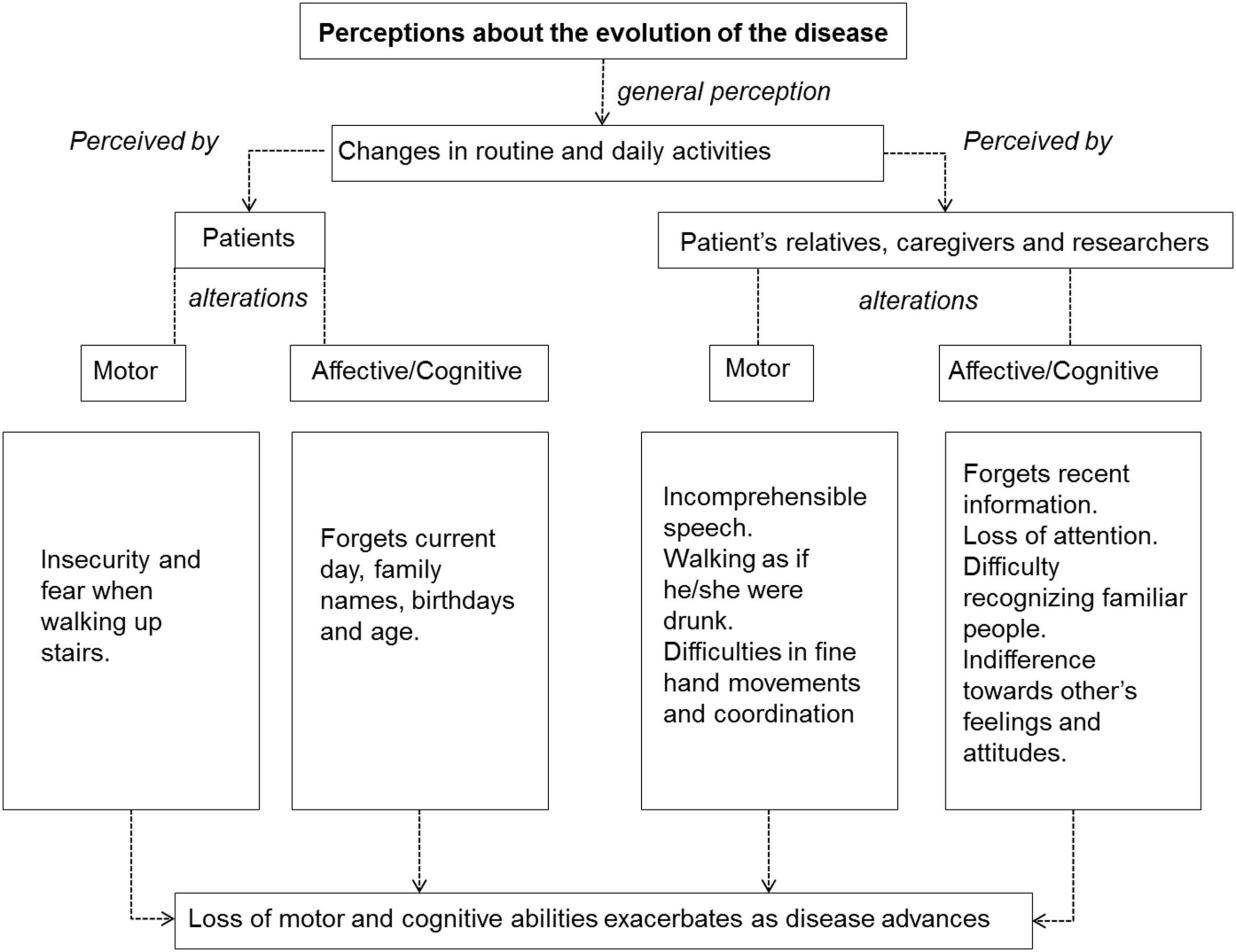
**Summary of the main participants’ perceptions while describing the evolution of spinocerebellar ataxia Type 2**.

A summary showing the main elements associated with the concept and evolution of the disease is presented in Figures 3 and 4.

When inquiring about the concept or definition of SCA2, it is common that individuals limit their explanations of the disease to the linguistic and motor deficits they have, in the case of the patients, or observe, in the case of the relatives. For example, when asked about the meaning of spinocerebellar ataxia some patients indicated:

Walking wrong, queasiness and problems when walking up the stairs. (patient P08)

It is the loss of movement in legs and hands. (patient P10)

Motor alterations were usually observed as the causes and not as the consequences of the disease. Patient P18 illustrates it as follows:

Once my child was sick and I took him to the doctor. When we arrived there in the taxi my child felt so heavy and I couldn’t carry him anymore. That’s why the disease began.

Some participants recognize the disease when they detect similar symptoms or movements previously observed in other ill family members. For example, the P25’s relatives indicated that they knew their sister was sick because she resembled her deceased father and brother:

She began to fall down a lot. They [ill people] walk as if they are drunk and lose their ability to move and then they can no longer walk. Then they get thin, they fade away. When her brother started he had cramps and spasms and she also has that.

Patients’ neighbors recognize the disease when they notice uncoordinated movements in the patients. This recognition can provoke either tolerance or support for the patients and their family, as in the case of people living in Tlaltetela and Mexico City. Also, it can provoke social exclusion since people are afraid of being infected, as it happened in Ezequiel Montes. For example:

For many years we were rejected in the town. We were outcasts because nobody wanted to be close to us. (P27, P25’ sister)

Spinocerebellar Ataxia Type 2 is a hereditary and non-infectious disease, and this is explained to the patient through an informed diagnosis mostly performed in the NINN in Mexico City. Nevertheless, patients frequently receive wrong diagnoses and treatments in the community health clinics close to their towns or neighborhoods. This situation can elicit disappointment and emotional frailty, which influences upcoming decisions about medical attention. For example, P23 indicated that “sometimes my mom was desperate and she used to say ‘I won’t go to the doctor anymore, they only give me vitamins. It’s waste of time and I don’t see an improvement.’ So, we decided to not go to the doctor any more.”

When the patients are properly informed about the SCA2 diagnosis, they explain their disease in neurological terms. Nevertheless, this kind of explanation is mostly given by the young patients or by the people living in Mexico City near the National Institute of Neurology and Neurosurgery (NINN). For example, when inquiring about the meaning of spinocerebellar ataxia, informed individuals indicated:

Some neurons are not working in my cerebellum. (patient P02)

My cerebellum is becoming smaller. It’s inherited. (patient P01)

It is a brain disease with no cure. (P03, father of patients P02 and P01)

When the family is properly informed about the disease, they become attentive to the symptoms. They go to the medical service regularly and sometimes decide to perform the predictive test to avoid the uncertainty and fear of having the disease themselves or their children. This was the case of the entire family living in La Moncada who decided to perform the predictive test when the hereditary causes of the disease were explained during the project. Also, it was the case of P27 (P25’s sister) who has a negative SCA2 test and dissipated her fear about her 9-year-old child whose nocturnal leg spasms made her think that he had SCA2.

Nevertheless, many years can pass before individuals obtain a correct diagnosis because many families cannot afford paying the trip to Mexico City. Besides, before being sent to the NINN, the patients have to be seen at several smaller health clinics, where medical personnel do not always recognize the disease. This was the case of patient P09 who was first diagnosed as a drug abuser, or patient P25 who was diagnosed with familial amyotrophic lateral sclerosis.

Another case is illustrated by P23 (P18’s daughter):

We took my mom to Toluca [State capital], then to the Adolfo López Mateos [a General Hospital in Mexico City] and they only gave her vitamins and calcium. What they gave us was not helpful and we don’t have enough economic resources to go regularly.

Difficulties to access a correct diagnosis and the ignorance surrounding the causes of the disease lead individuals to look for alternative treatments. These treatments can be based on traditional folk medicine even if they have to spend a great amount of resources. This was the case of the family living in Tlaltetela and the family living in Coatepec.

P22 (P18’s husband) illustrates this situation:

I took her to ‘curaciones’ (healing) and ‘limpias’ (cleansing) performed by traditional healers. They told me to bring roses and some candles with me. We spent 1.700 pesos.

Individuals identify the beginning and the development of the disease through fear or insecurity elicited by the loss of movements that limit their everyday activities. These are some testimonies about how the participants realized they had the disease:

I felt afraid when walking and I had to grasp everything. (patient P25)

My feet trembled and it was difficult to use stairs. I felt exhausted all day long and I realized that I was sick like them [her father and her sister]. I still can cook and wash my clothes, but I can’t make tortillas anymore. (patient P07)

I am afraid I might fall when taking a shower. (patient P11)

Some alterations are not referred to by the patients until they are mentioned and observed by others. This situation was observed when applying the psychometric test during the study since patients could not hold the pencils to write or touch the computer screen, even though they indicated they could solve the test without any problem. Alterations in walking and speech are other examples since patients do not realize their difficulties until others indicate they act as if they are drunk. For example:

My mom used to work in customer service in an office. People complained about her incomprehensible language and they said ‘the lady is a little drunk’. But my mom doesn’t drink and never did. (P05, P04’s son)

Many people told me I was drunk, even in the morning. I always had to convince them that I wasn’t drunk. (patient P17)

Only a few patients expressed having cognitive alterations, such as, lack of memory or difficulties in attention. Nevertheless, it was common that they forgot episodes or people during the interviews, such as, the name of their neighbors, places, their birthday, or their children’s ages. For example, patient P19 got confused when talking about his children’ ages and names, patient P25 indicated we were living in 1914, or when asked about his brother’s age patient P09 indicated “I don’t remember. I could before but not anymore.”

Difficulties with retaining attention, loss of memory, and an inability to recognize known faces were indicated only by the patients’ non-sick relatives. For example:

She doesn’t think well since one or two years ago. She used to take care of the family business and she did it very well. She was great for calculation and money operations. People used to say she had a very good memory. But now the information can’t be kept in her mind. (P26, P25’s mother)

Family often refers to hostile behaviors such as a lack of social conventions and indifference to other’s feelings. For example:

Once my husband was scratching my sister. When they [patients] get angry they hold someone and don’t let him go. When we walk beside her [patient P25] we get bruises [in the arms] because she grabs us. She doesn’t want to do anything and she is so mulish. (P26, P25’s mother)

Imagine that we have three little birds here. They only open their mouth and they know that they will have everything. They don’t care how the things [food, clothes, or money] arrive home. They don’t reason. (P03, P01 and P02’s father)

Some patients perceived they were sick because of changes in their working, affective, or interpersonal routines. For example:

I am sick because I used to play soccer and at age 15 I told my father I didn’t want to play anymore. I can’t move like I used to. (patient P02)

She quit her job because of the problems in her speech and communication. She used to cook a lot and she was taking care of her home all the time, but she can’t do it now. She abused the attention we gave her but she tries to perform her activities as normally as possible. (P05, P04’s son)

### Feelings and pain

**Figure 5:**
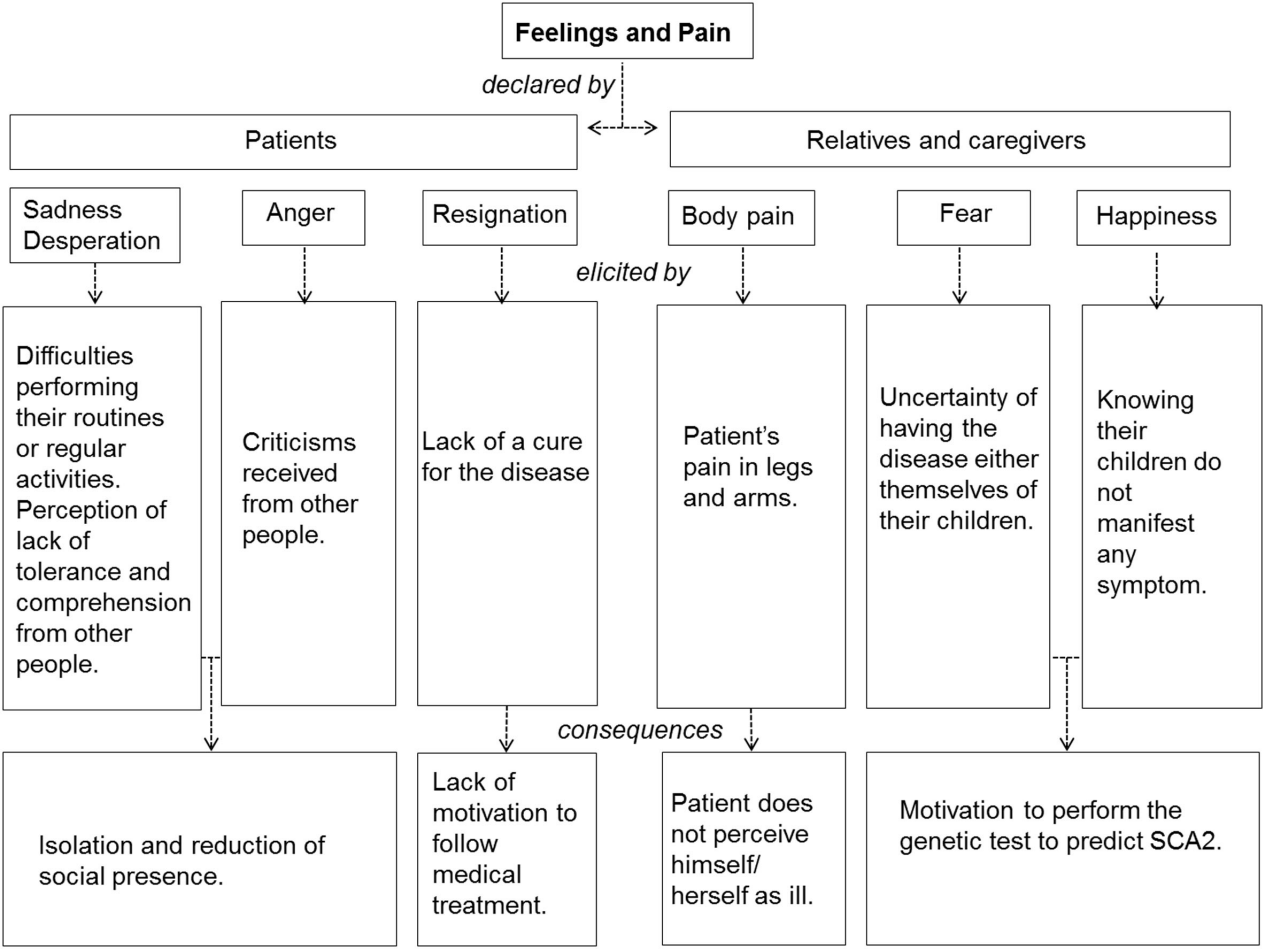
**Summary of the main participants’ feelings declared while performing the interview on their experience with spinocerebellar ataxia Type 2**.

A summary for the main feelings and pain expressed by the participants is presented in Figure 5.

The patients do not express feeling pain even though their medical files mention muscular deterioration and numbness. This is one of the reason they think they are not sick. When pain was referred to in the interviews, it was mentioned only by their non-symptomatic relatives. For example:

It is true that I’m not doing so well and I have seen other patients who are not healed. But for me it isn’t a frustrating disease. I don’t feel pain and the only difficulty I have is walking when it’s cold. It mostly affects me economically, since I realize that I can’t work as before. (patient P17)

Declarations about the patient’s feelings were confusing and may be attributed to low vocabulary and educational levels. Also, it can be attributed to the typically Mexican social formation, which restrains emotional expressions associated with weakness in men. For example, when inquiring about how the patients felt when they knew they had SCA2, patient P08 indicated “I’m doing badly. [Estoy mal.]” a Mexican expression that does not refer to physical malaise but emotional discomfort. When asking P08 about specific feelings and emotions, such as, anger or sadness, he indicated that “the most similar emotion is sadness because I will not be able to do many things, such as working the fields.”

In some cases, resignation was declared as an emotion elicited when watching their sick family members or their family members that have already died. For example, patient P09 indicated “I am resigned because my dad died like that. I have been resigned from the beginning, but I am sick so, it doesn’t matter.”

Sadness and resignation are associated to changes in routines and losing social presence. In this sense, patient P07 indicated: “I am truly sad because I will not do the same things I used to do.” When asking her about how she faces the situation and the duration of the feeling she indicated: “I feel sadness all day long. Then I feel better by myself. I just try to keep the sadness away since I am sick anyway.”

Patient P09 indicated:

I just cry at night when I remember my husband who is already dead and can’t help me anymore. I am alone at home all day long but I go to the kitchen to sit in the chairs and wait until my children arrive home.

On the other hand, feelings of anger were associated with criticisms arising from people that are not close to the patient or their family, which make their social inclusion more difficult. Regarding this, patient P07 pointed out that “sometimes they (people) tell me I don’t walk well” and patient P14 said, “Anybody get me a job.”

In contrast to the resignation and anger manifested by these patients, non-symptomatic relatives reported feeling fear. They expressed feeling afraid of having the disease themselves but also that their children might have it. For example, P27 (P25’s sister) indicated:

I am afraid when I think about the disease. When my son is sleeping he has spasms and they [her brothers] started like that. So, I am afraid my son will be sick too.

Non-symptomatic relatives are motivated to perform the genetic test when they learn about the heritable mechanism in SCA2. These were the cases of the families living in La Moncada, in Ezequiel Montes, and in Coatepec, who decided to perform the test after we started the interviews with their families. In this sense, P16 living in La Moncada said:

Those who don’t have symptoms talked about the disease and I feel that we have to be psychologically prepared. We have to know the indications we need to follow if we have the disease as if we were diabetic we would have to take medication for the entire life. We have to be aware that in order to have a normal life we have to follow medical advice. We have talked all about it. If we know that one of our children has the disease we have to support them and make him or her understand that not he, she, nor anybody else is guilty about it.

Nevertheless, the motivation to know the disease’s mechanisms is not enough since several personal and cultural variables influence the decision to have the test done. P24 (P18’s daughter) illustrates this point:

The test is a good option to avoid the disease, to avoid its proliferation. But, at the same time I wouldn’t like to have it done because I want to have a baby.

Hence, the test is controversial since the results can affect previous plans based on their cultural roles. P24 (P18’s daughter) mentioned:

They [medical personnel] informed us about the test and asked if we wanted to know who could inherit the disease. We had the opportunity. I said that to my brothers but they said ‘No!’ because if they perform the test they will be disturbed. The elder is living with a woman but I think they aren’t considering children. I hope God doesn’t want them to be sick. Similarly, my sister said she will only have two children because they might have the disease.

Although feelings and emotions were topics stated during the interviews, these elements are not commonly manifested within the family in most of the cases. Anger, sadness, and other feelings are regularly experienced in silence and in a lonely way. An extreme case was manifested by P03 (P02 and P01’s father) when he expressed that he has thought about committing suicide in order to avoid his family’s suffering, but his Christian beliefs have kept him from doing this. Another example is illustrated by patient P18, who stated:

I felt desperation and I would like to live as I used to. But I can’t, so they [family members] reprimand me sometimes. I would like to go far away where I don’t cause any disturbance for anyone.

Positive feelings, such as happiness and hope were mentioned when non-symptomatic participants knew that they or their children have a negative diagnosis for the disease. For example:

I feel good when I see my sons and grandsons are fine. (patient P11)

### Social and institutional support

**Figure 6:**
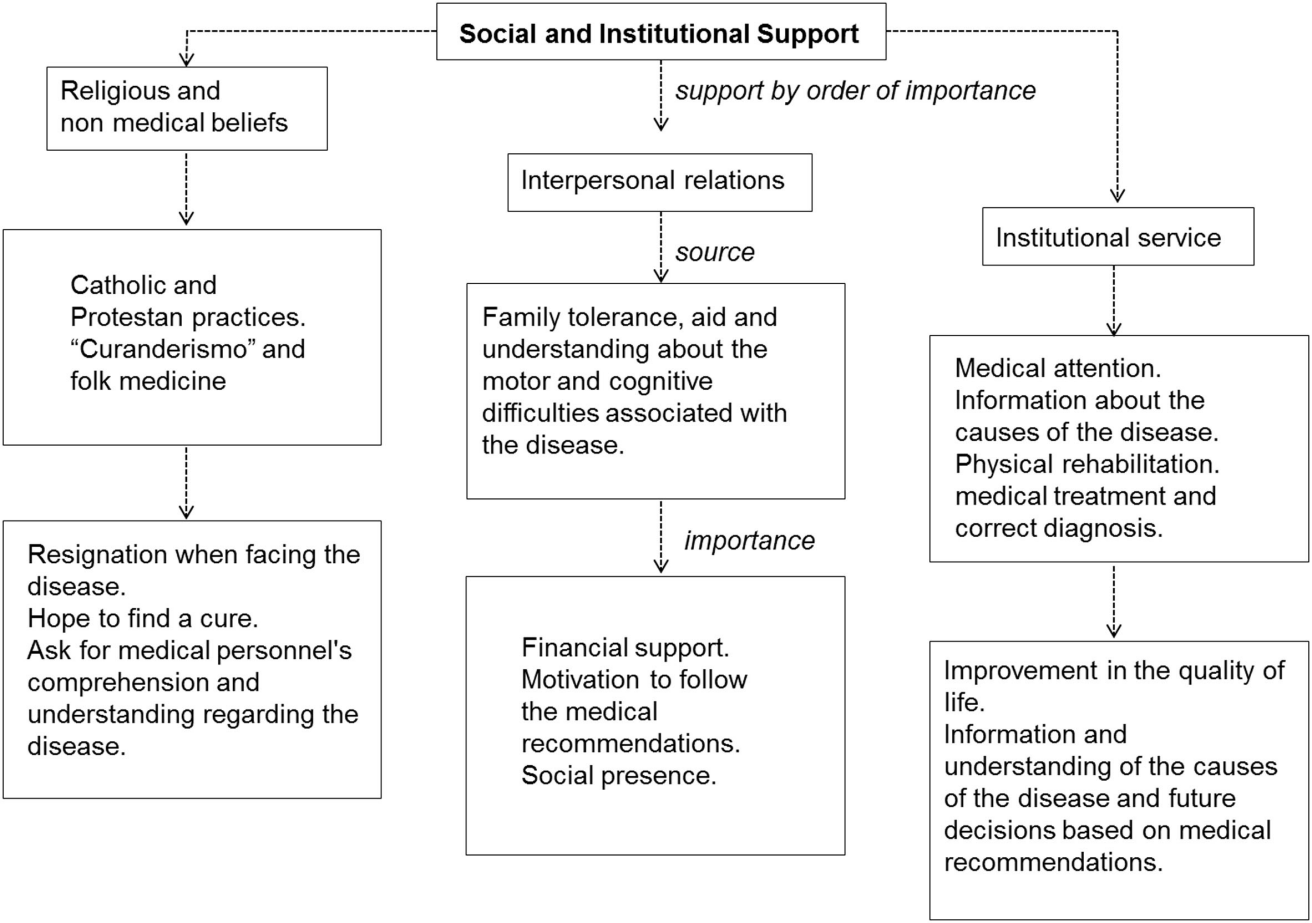
**Summary of how the participants’ perceive support when dealing with spinocerebellar ataxia Type 2**.

Participants’ mainly perceived support when facing the disease comes from three components in the following order of importance: religious beliefs, interpersonal relations, and medical institutional services. A summary of how the participants’ perceive support when dealing with the disease is presented in Figure 6.

The support provided by their religious beliefs is observed in prayers asking for motor alterations to end, although not for a cure of the disease. For example, from his Catholic perspective patient P08 asks for “control” referring to his uncoordinated movements.

Even if some patients consider a divine intervention in the disease this intervention is not qualified as negative. For example, patient P07 indicated that “I do not blame him (God) for anything; I have to continue living my life.”

Prayers also include worries about their children in the case of women, as is illustrated with patient P11 who asks God that “I hope not to be absent for my sons.”

In the case of men, Protestant and Catholic prayers are directed toward having the medical personnel understand the disease. For example:

I always pray for the doctors, nurses and laboratories and everything that refers to health. I ask God to give them wisdom and knowledge and everything they need to save a person who God hasn’t asked for. (P03, P02 and P01’s father)

Some patients indicated that their beliefs in God became stronger not when the disease appeared but as the social and economic consequences arose. As patient P14 indicated “it is true that we need to have somebody we’re attached to.”

The second source of support is the family where patients find tolerance to do their daily activities slowly, to receive attention and not to be criticized because of their wrong movements and speech. As P24 (P18’s daughters) indicated “we only let her wash the dishes and help prepare the food, we tell her ‘do your things slowly, nobody is rushing you, we’re here with you’.”

The family support becomes more complex and difficult when other members of the same family present the disease too. For example:

Sometimes they [his family] ask me if I need help and I need it, so they help me to load my bag. But they can’t help much because they are like me and I can’t help them. (patient P17)

Family relations are important for learning several different ways to face the disease. In this sense, P12 indicated that:

I am around here constantly just in case she needs something (his sister P09), if she needs to move somewhere or if she has any issues with her children because her husband is already dead. The last time her daughter (patient P10) became a little rebellious and she said ‘I think my mom doesn’t understand me’. So, I told her she has to be understanding since her mother may not understand very well due to the disease, so she has to be tolerant.

Family support is fundamental in the patients’ emotional sphere to motivate them to attend constant medical revisions. If the family is not persistent the patient loses his motivation to continue with the medical treatment, which may improve the patient’s quality of life even if it does not represent a cure and provide more information on the disease. Patient P10 illustrates this point when she says that “they (her family) have helped me to prepare and eat meals that I did not use to eat. I eat vegetables now, but I used to eat only sausages before.”

Financial income is the most important element within family support because most of the patients cannot work. This is particularly relevant in our participant group when considering their low economic resources. Economic disturbances are worse when the patient is the family head and must provide for their family, as is the case of patients P19, P14, and P17. In this situation, discrimination concerning the disease in some towns and places keeps patients or their relatives from being integrated in local economic activities because people fear they are contagious. This is the case of the family living in Ezequiel Montes where the sisters who did not suffer the disease migrated to another city to find a job and send money to their mother and sick sister to provide for daily and medical needs. This case is remarkable since this family is the only SCA2 focus in the town but the father, who was the first case, came from a small village close to Ezequiel Montes where, as the family indicated, many sick people live and they had not been identified by the public health system.

Extended social networks are important cultural elements for family integration in the local and regional productive life. The neighbors’ and inhabitants’ attitudes move from tolerance and comprehension, as it is the case of Tlaltetela, to exclusion as in the case of Ezequiel Montes. When asked about the way they think other people perceive them, participants indicated:

We only see they look at them as if they were odd creatures. (P24, Coatepec).

People ask me about my situation and I tell them I have a head disease and that is all. (patient P07, La Moncada).

We were stigmatized when we went to school, as if we were nauseating. Nobody wanted to be near us. (P27, Ezequiel Montes).

Generally, the public health service is considered as the last support and is perceived as inefficient and disorganized by most of the participants when referring to access to medication. The perception of efficiency improves when the participant lives close to the NINN and/or has economic resources favoring the patient’s mobility. This was the case of patient P04 whose medical file is the most complete and included monthly psychiatric and neurological evaluations and attention. Her son (P05) said that:

I think it’s a good system because you have a doctor and when she had a crisis she received attention. We were in the ER almost every day and they gave her medicine. They always offered a good service. But it was familiar processes because our family was involved and they have our family tree. Then my brother and I performed the predictive test. We are negative.

As the last testimony indicates, the NINN includes informative meetings for patients and their relatives, where the responsibility of the family is evident. Nevertheless, this monthly orientation program was suspended due to the frequent absence of the patients. Some small health community centers outside of Mexico City are informed about the patient’s diagnosis either by the NINN personnel or by the patient himself. When this is the case, the center may perform its own empiric programs for muscular rehabilitation to keep the patient’s mobility as optimal as possible. For example, patient P10 used to go to a small rehabilitation center in Tarimoro, the Municipality Head of La Moncada, and she reports that: “they make me do exercises with my arms and legs, then with a bicycle to have better mobility in my legs. But I could not go anymore because the sessions were so early and then I had to go to school.”

Difficulties when accessing the institutional medical service due to lack of economic resources or a lack of knowledge concerning the disease lead individuals to look for alternative attention. These alternatives depend on economic resources and beliefs, as in the case of participants in Coatepec who went to “curanderos” [traditional healers], or the P04’s family who use popular nutritional supplements. For example:

She tells me when the medicine is over. Now we can find Q10 here in Mexico, but before we had to look for it in the United Sates. That is a medicine we can buy with any medical prescription and we can find it in WALMART. It is used for oxygenation. They recommended we go to hyperbaric oxygen therapy but they were expensive so, we couldn’t go regularly, so we researched about it and we found the Q10 pills (P05, P04’s son).

In municipalities far away from Mexico City access to the medical institutional service is commonly perceived as dependent on political parties’ interests and not on the rights to be assisted. As patient P14 indicated:

Look! We went there [to the medical service in the town] with my girls in the past years. But as you can see we have a new party in the government so they asked us to become a part of it. We refused, so they don’t receive us anymore. They have only seen us four times since then.

The lack of a cure for SCA2 and the anger and frustration associated with it elicit judgments concerning the medical service. Also, it influence the patient’s and their relatives’ responsibility when following medical suggestions. For example, P03 (P02 and P01’s father) said:

Once I said to a doctor, ‘if you don’t do anything for my family I won’t bring them anymore’. He told me that it was an insult and that the hospital doesn’t need our money. He also told me it was my responsibility to go to the hospital and if anything occurred to my children I can’t say that it was the hospital’s responsibility. On the one hand he was right but on the other I was right too, because I do not see any improvement with the medicine. I only want them [the patients] to be able to feed themselves and not to have to pay for diapers. If they are doctors they have to know, in the same way that if I am a chef I have to know the amount of salt needed to prepare a dish. They can follow the recipe book. That was how I though before, but I am resigned now. Only a miracle from God can solve the situation.

## Discussion and Conclusion

One discussion that can be derived from the ethnographic records is the relationship between the individual’s perceived reality and the cognitive and brain deterioration typically manifested in SCA2 patients. To relate these elements, we will consider the VBM results of the 15 patients participating in this study that show a deterioration in the cerebellum and brainstem, as well as in insular, frontal, temporal, and parietal cortices (60) (see Supplementary Material).

The more significant deterioration in our sample was observed in the brainstem and in the uvula of the vermis, located in the lobule IX of the posterior cerebellar cortex (60). Typically, deterioration in these both regions is observed in SCA2 patients (13, 71, 72) and may be associated with motor alterations and lack of coordination clearly manifested by the participants and reflected in the SARA. Nevertheless, besides the motor aspects, the vermis plays an important role in regulating emotional expressions and inferring others’ emotions (73) through a network involving other cerebellar regions, such as the flocculonodular lobe, the fastigial nucleus, and, the globose nucleus, as well as subcortical limbic regions (74, 75). The role of the vermis in SCA2 should be studied in a more accurate manner in future research since its damage is associated with the cerebellar cognitive affective syndrome and with emotional fragility and alterations in facial recognition in several SCA types (25, 29–31). The emotional role of the cerebellum has scarcely been considered in neurocognitive analysis for SCA2 patients. Nevertheless, its atrophy may be related to the emotional indifference and hostility perceived and reported by the testimonies of the patients’ relatives in advanced stages. This future route is interesting since the cerebellum may acts as a predictor of autonomic responses during emotional experiences along other brain regions observed as atrophied in our participants, such as, the middle temporal gyrus, the frontal cortex, and the insula (76).

As it may be observed in the participant’s testimonies, SCA2 affects not only the execution of movements but also their planning, which may be linked to cerebellar deterioration since its function is related imaginary movements or the preparation of complex actions (77, 78). These motor and sensorial mechanisms may influence the patient’s own body image and identity, both determined by the dynamics of experiences and learning (79) and the capacity of movement (80). Therefore, body image is not only a passive perception but an active process. The loss of movement in SCA2 is relevant to understanding both the patient’s social presence and the importance given by the participants to define and understand the disease according to their mobility.

Language disorders affect patients’ ability to solve the psychometric test traditionally used to evaluate these patients, such as the MMSE and the MoCA test, as observed during the study. These disorders also imply the urgency to recover speech to express their psychological states and be socially inserted and accepted, as it has been observed in patients with other types of degenerative ataxias (55). Language difficulties may be related to the deterioration in the premotor cortex and inferior frontal, temporal, and pariteal cortices (19, 71, 81). These deteriorations not only affect high motor sequences but also the production and comprehension of their own and others’ speeches (82–84) constituting a fundamental element to be considered in SCA2 and other SCA types (85).

The insular deterioration observed in these patients may be related to muscular and motor alterations (16) as described in the ethnographic results and in the clinical reports of patients. The insular cortex has barely been considered in theoretical models of SCA but may represent a central brain region. The insula maintains anatomical and functional connections with frontal and temporal regions that are also deteriorated in these patients and are needed to perform goal-directed actions correctly. It is also important to process the interoceptive information needed to represent body topographies influencing empathy and social cognition (86, 87). Insular deterioration may be related to divergent patients’ body images inferred from the lack of awareness of their motor alterations until somebody else indicates them, such as, incomprehensible speech or difficulties in writing while performing the psychometric test. This may also be related to the emotional indifference or lack of empathy (23, 88, 89) manifested by the relatives when living with the patients.

The social, cognitive, and emotional alterations exposed in the ethnography may also be influenced by the deterioration in the temporal pole observed in these patients (60). The function of this region is related to the attribution of social and emotional qualities and actions in others (90). So, the understanding of the disease involves social and emotional qualities assigned to the negative consequences of the disease that have to be take into account to explain it. Also, this understanding can be used to design psychological support programs and neuroimaging functional studies to evaluate brain related cognition.

The parahippocampal gyrus was identified as atrophied in the VBM analysis for the patients participating in our study (60). Since the function of this region is associated with long-term memory formation in social environments (91, 92), its atrophy may be related to the participants’ testimonials indicating alterations of memory and difficulties in learning new social abilities to face the disease (22).

Clinical evaluations and psychometric measurements in SCA2 patients mainly involve motor skills. Nevertheless, there is a vast diversity of emotional and social aspects affected in this disease (13). So, research on SCA2 should consider these multiple aspects to understand the patients’ mobility in their cultural environments, which may influence their concepts about the disease and the attachment to medical treatment (17, 67).

On the other hand, from some anthropological and ethnographical perspectives, suffering from chronic diseases, particularly those that affect motor aspects, implies the loss of self since the individual is isolated and socially disparaged (50). Isolation affects self-esteem and social relationships (55). This is why the support provided by the family and other people close to the patient become fundamental to maintaining the quality of life and social presence of SCA2 patients.

According to other research and the participant’s testimonies, patients’ families represent a source of resources that help solve health related problems in SCA2 patients (93). Family relationships influence and, in some cases, may determine the level of evolution of the disease, the perception, and the ways of facing physical and motor consequences. The presence of the disease in a member of the family implies important changes in the family’s routine, the re-organization of personal lives, and the emergence of some emotional traits such as, anxiety, emotional instability, and economic needs. However, these elements have scarcely been considered in the diagnosis and medical continuation and may indicate notorious inequalities for the individual’s access to the public institutional health system.

Family dynamics are particularly important in accepting predictive tests for SCA2 and in evaluating the consequences. As we observed, main reasons to accept the test are the possible alleviation of the uncertainty, the notion of a control over the future and the decision to reproduce. In this context, several emotional traits such as anxiety and depression can emerge among family members (94). As inferred by the participants’ testimonies, these elements are not considered in the health system perspective. These emotional traits can be a reason to reject the predictive test along with cultural variables that demand reproduction as a familiar identity where patients live. Although each family was different, there are general structures or unities depending on several subsystems in which the patient is the minor unit that receives medical treatment (95). So, not only must the patient be considered but also the family must be part of the medical research surrounding SCA2. A methodological alternative could involve the parental ethnotheory focused on the ideas that parents build around their family (96) and frame patients’ experiences in the collective history of their community.

As observed in our study, the presence of SCA2 demands an excessive dependence between family members, which may restrict personal growth and achievement. The therapeutic approach to SCA2 could be directed to design a flexible family system to change rules and roles for conflict resolution. This may easily exemplify many cultural roles that fall on the individuals, such as gender roles in Mexico where women are attributed as the caregivers, men are restricted in expressing their emotions, and pain and suffering are considered as good qualities (97, 98). This was observed in the study through the difficulty of finding verbal emotional expressions among patients living far from large urban places.

Spinocerebellar Ataxia Type 2 affects the patient and their family’s identity as healthy people in their community. The heritable quality of the disease also affects the non-symptomatic members because they share the social consequences suffered by the patients (17). In this sense, it could be interesting to consider a type of parental attachment, understood as links established between fathers and sons to understand future alterations in emotions or difficulties in recognizing emotions in others (99). Emotional difficulties can emerge as consequences of SCA2 in the parents as it was showed in patients with children. Considering these elements may improve the psychological support in the treatment and the effects of the predictive test which is becoming more important in these rare diseases. Also, it may be useful to understand that the decision is socially situated and based on notions of responsibility with deep implications for the family as a system (100–102).

With regard to the relation of the patients with the public health system, the Mexican tradition of “curanderismo” (traditional healers) must be considered as a culturally legitimate option for alleviating illness, particularly those involving mobility and muscular skeletal conditions (103, 104). As manifested in the results, the use of “curanderismo” and other non-scientific medical beliefs play an important role in alleviating a sickness with no medical cure. Moreover, it is important in understanding the disease and facing its emotional and social consequences, for example, through resignation. It is by means of these beliefs that the patient and their relatives elaborate causal explanations for the disease out of the conventional medical approach, such as, living wrongly or viewing pain as a redeeming quality influenced by the Catholic culture prevalent in Mexico. Therefore, from an anthropological approach the health-sickness process considers beliefs as a result of needs that are not satisfied and sometimes are not perceived. In general terms, this process reflects the living conditions of a population and the dependence of the economic structure and social organization of the country (51). Cultural differences regarding health between countries and socioeconomic classes are important because, as shown in our study, they are the expression of the organic, psychic, biological, functional, socio-affective, and collective harmony which favors a more productive life.

This final idea is important for understanding social and cultural aspects associated with SCA2 since the language used in the public health system campaigns may not been understood by the social groups who suffer the illness. This is illustrated in the diverse conceptions of the disease manifested by the participants and based not on the causes but on the symptoms. It seems that problems of communication exist between the health system, the professionals and the services offered, as occurs with rare diseases. These problems can misinform the patient and influence the cognitive dissonance when patients know that people in charge of curing the disease have no access to the cure, but continuous medical care is needed (56). Besides, as presented in the SCA2 patients, most of the patients who participate in research hope for useful short-term results when faced with the complexity of the disease (55).

Although health can be equivalent to vigor, the ability to work, and capacity to respond to a social request, this concept is a cultural interpretation and far from strict medical knowledge (51). This concept influences some reactions to the disease, such as, the social rejection and exclusion since people with SCA are not considered to be productive. Another relation between culture and health is the notion that a habit of balanced nutrition and exercise is needed to stay healthy, but these are not natural orders as understood from many medical biological approaches. Indeed, they depend on varying economic and educational resources between populations and particularly important in our SCA2 sample which involves low educational levels and economic resources.

Health systems focus mainly on direct or immediate solutions, but in the case of SCA2, a priority should be changing culture and attitudes toward health and how to coordinate patients’ behavior and the people around them in environments outside of the medical institutions (105). In this case, the participation of the patient is fundamental in order to know the public health system’s actions and effects. For example, according to similar reports (55), patients in our study perceive null or scarce psychological support and some of them have to look for alternatives for physical rehabilitation and medication outside of hospitals. Biomedical sciences should consider that even SCA2 patients receive medical follow up, their insertion depends on whether or not their social group maintains its own perceptions, emotions, and culture. This elements may contribute to the experiential knowledge of the disease and therapeutic alternatives (106).

The joint analysis based on neurocognitive and cultural representations of SCA2 contributes to a medical anthropological perspective. This perspective may bring together genetic, clinical, and social aspects to refine the diagnosis and the treatment based on experiences, as has been the case in other congenital diseases (102). The motor and bodily aspects most deteriorated in SCA2 involve mobility in social, symbolic, and health spaces, which influences the individual’s responsibility over their own body and beliefs or their justifications for isolation, inaction, and conformation (107).

It may become necessary to develop an organizational structure of research in neurosciences and health based on interdisciplinary views where cultural experiences and social methods complement experimental findings. It is uncommon for biomedical research to consider the patient’s own knowledge because it is considered subjective. Nevertheless, as our work shows, narrative is a way of accessing personal descriptions using metaphors and images that model patients’ and others’ experiences. In turn, this allows researchers to understand the patients motivations, consciousness, and emotions that would remain unknown in any other way (108). The knowledge transmitted by the patients and their relatives can be translated as working hypotheses involving the progression of the disease, or the design of different therapeutically approaches. For example, we can formulate hypotheses on the role of the insula and the cerebellum in the patients’ motor cognition and consciousness, or the role of insular-frontal networks on the inference of social emotions, as proposed for frontotemporal dementia (109). Also, our findings can be used to locate geographical distributions of SCA2 through informant social networks not inscribed in the institutional databases as it was the case of Ezequiel Montes, or to observe tolerance toward the disease as it was the case of Tlaltetela where a high prevalence of SCA is reported (59). This also contributes to developing more accurate ethical principles for voluntary and long-term participation, informed consents, and predictive tests.

By bringing together culture, brain function, and neuropsychiatric diseases, a social neuroscience perspective is born where the borders among psychology, anthropology, and neuroscience overlap. This approach considers the individuals’ cultural situation within various geographical spaces, but maintains an equivalent biological history that is accessed throughout their cognitive manifestations. Usually, the researcher interprets the patients’ perceptions through his own cultural codes, which can be different than those prevailing in the patients and their family. Accordingly, as stated in our objectives, ethnographical methods complement neuroimaging and psychometric evaluations to understand SCA2. The ethnographical approach illustrates the other’s perspective in his context, in his daily experience and through his own voice and behavior. Furthermore, it allows them to feel closer to the researcher and leads to a dialog involving his condition. Although the experience of SCA2 in patients and researchers is different because they come from different contexts, the possibility of a dialog and a new shared experience lead to a better understanding of the cultural and natural aspects of this disease.

## Author Contributions

REM conducted and analyzed the ethnographic interviews, obtained the participants’ resonance magnetic images, interpreted data, and elaborated the text, tables, and figures; VG and RD elaborated the institutional database to access the patients and their family and revised the clinical files; LP analyzed and interpreted the ethnographic interviews; JV-M interpreted the behavioral data and elaborated the discussion; CH-C realized psychometric evaluations and analyzed behavioral and imaging data; JF-R leaded the research on SCA types in Mexico, interpreted imaging and behavioral data, elaborated the text, tables, and figures.

## Conflict of Interest Statement

The authors declare that the research was conducted in the absence of any commercial or financial relationships that could be construed as a potential conflict of interest.

## Supplementary Material

The Supplementary Material for this article can be found online at http://journal.frontiersin.org/article/10.3389/fpsyt.2015.00090/abstract
