# Supplementary material for: Social and Cultural Elements Associated with Neurocognitive Dysfunctions in Spinocerebellar Ataxia Type 2 Patients
Source: Front Psychiatry. 2015 Jun 10;6:90. doi: 10.3389/fpsyt.2015.00090 (PMC4462049; doi:10.3389/fpsyt.2015.00090)
Supplement: Supplementary file 1 [file datasheet_1.pdf]

Supplementary Material

Social and cultural elements associated with neurocognitive dysfunctions in Spinocerebellar Ataxia Type 2 patients

Roberto E. Mercadillo[1,2,3], Víctor Galvez[4], Rosalinda Díaz[1], Lorena Paredes[5], Javier Velázquez-Moctezuma[3], Carlos R. Hernandez-Castillo[2,6], Juan Fernandez-Ruiz[1, 4, 7]*

[1] Laboratorio de Neuropsicología, Departamento de Fisiología, Facultad de Medicina, Universidad Nacional Autónoma de México, Mexico City, Mexico.
[2] Consejo Nacional de Ciencia y Tecnología-Cátedras, Mexico.
[3] Área de Neurociencias, Departamento de Biología de la Reproducción, Universidad Autónoma Metropolitana, Unidad Iztapalapa, Mexico City, Mexico.
[4] Posgrado en Neuroetología, Universidad Veracruzana, Xalapa, Veracruz, Mexico.
[5] Facultad de Psicología, Universidad Nacional Autónoma de México, Mexico City, Mexico.
[6] Instituto de Neuroetología, Universidad Veracruzana, Xalapa, Veracruz, Mexico.
[7] Facultad de Psicología, Universidad Veracruzana, Xalapa, Veracruz, Mexico.

*** Correspondence:** Dr. Juan Fernandez-Ruiz. Departamento de Fisiología, Facultad de Medicina, Universidad Nacional Autónoma de México, UNAM, Coyoacán, Distrito Federal, México, 04510. Phone: (52 55) 56232393. E-mail: jfr@unam.mx

1. Supplementary information about the Voxel Based Morphomety data.

The brain regions identified as atrophied in the Voxel Based Morphomety analysis were previously obtained in the 15 SCA2 patients participating in this study contrasted with 15 control matched participants. Details of the methodology and discussion are presented in Mercadillo et al., 2014.

Images were acquired in a 3 Tesla Philips Achieva MRI scanner (Philips Medical Systems, Eindhoven, Netherlands), National Institute of Psychiatry, Mexico City. A high-resolution 3D volume consisted of a T1 Fast Field-Echo sequence, with TR/TE=8/3.7 ms, FOV 256×256 mm^2 and an acquisition and reconstruction matrix of 256×256, resulting in an isometric resolution of 1×1×1 mm^3.

To analyze grey matter reduction we used VBM (Good et al., 2001) implemented on FSL and converting the original DICOM files into a NII format (Smith et al., 2004). Voxels belonging to neck and other non-brain tissues were eliminated using the BET software and grey matter was segmented before being registered to the MNI 152 standard space using non-linear registration (Andersson et al., 2007). The images were averaged and flipped on the x-axis to create a symmetric, study-specific grey matter template. Owing to the non-linear component of the spatial transformation, original grey matter images were then non-linearly registered to this specific template and modulated and corrected for local expansion or contraction. The modulated grey matter images were softened by applying an isotropic Gaussian 3 mm kernel. The voxel-wise GLM was applied using a two-sample t-test corrected for multiple comparisons across space. The voxels considered as significant differences between groups present a cluster-based correction at $p < 0.05$.

2. Supplementary Tables

Brain regions identified as atrophied in the Voxel Based Morphomety analysis previously realized in the 15 SCA2 patients participating in this study contrasted with 15 control matched participants.

2.1. **Supplementary Table 1.** Neuroanatomical regions indicating a significant decrease of grey matter volume in the 15 SCA2 patients (Table modified from Mercadillo RE et al. 2014. *J Neurol Sci* 347(1-2):50-8.)

Lat.	Brain region	Anatomical location	Brodmann's area	Cluster Size	Coordinate x	y	z	1-p (corrected)
L	Frontal lobe	Precentral gyrus	4	40	-30	-28	64	0.982
L	Frontal lobe	Middle frontal gyrus	6	222	-24	-6	48	0.989
R	Frontal lobe	Inferior frontal gyrus	45	285	34	24	8	0.996
R	Parietal lobe	Inferior parietal gyrus	40	4	-52	-28	28	0.985
L	Temporal lobe	Middle temporal gyrus	38	32	-36	4	-38	0.980
R	Sub-lobar	Insula	13	681	-40	-32	22	0.992
L	Sub-lobar	Insula	13	991	-34	24	8	0.998
R	Limbic lobe	Parahipoocampal gyrus	28	6	22	-12	-8	0.985
L	Limbic lobe	Parahippocampal gyrus	28	181	-20	-14	-14	0.995
L	Brainstem	Pons	*	6	-20	-22	32	0.977
L	Post. Cerebellum	Uvula of vermis. Lob. IX	*	20680	0	-66	-34	1

Note. Coordinates represent the peak value of the cluster in accordance with the Neurological Institute of Montreal (MNI) template. Approximate Brodmann areas were obtained through the Talairach Daemon System. * Indicate no Brodmann's area reported.

1. Supplementary Figures

Supplementary Figure 1. Sagittal, axial and coronal slices showing cortical regions with significant gray matter volume reductions in SCA2 patients. Green crosshairs indicate the local maxima differences. A. Left precentral gyrus (BA 4), B. Left middle frontal gyrus (BA 6), C. Right inferior frontal gyrus (BA 45), D. Right inferior parietal gyrus (BA 40), E. Middle temporal gyrus (BA 38). BA= Brodmann's area; S= superior face; I= inferior face, A= anterior face; P= posterior face; R= right face; L= left face (Figure modified from Mercadillo RE et al. 2014. *J Neurol Sci* 347(1-2):50-8.).

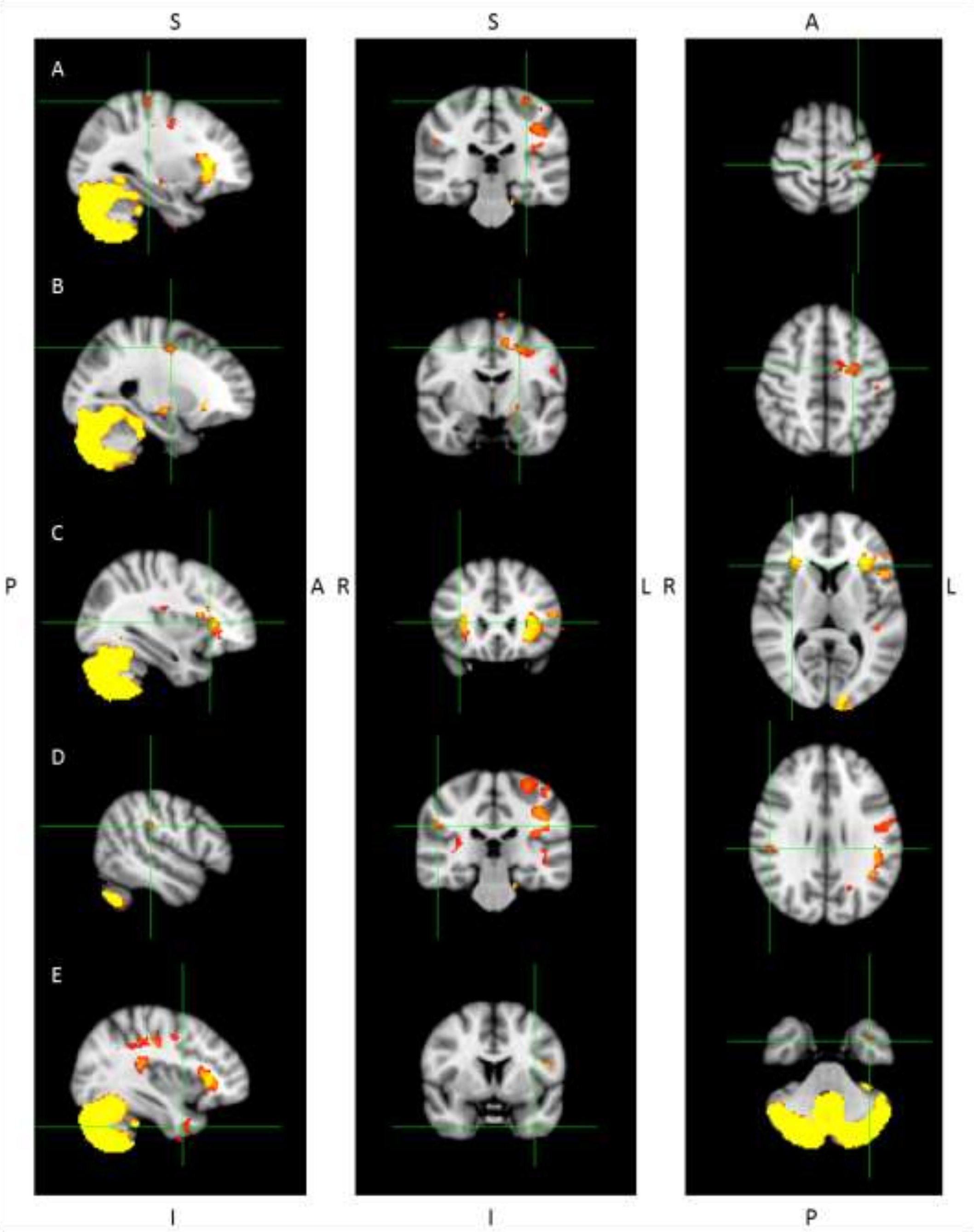

Supplementary Figure 2. Sagittal, axial and coronal slices showing subcortical and cerebellar regions with significant grey matter volume reductions in SCA2 patients. Green crosshairs indicate the local maxima differences. A. Right insula (BA 13), B. Left insula (BA 13), C. Right parahippocampal gyrus (BA 28), D. Left parahippocampal gyrus (BA 28), E. Left brainstem (pons), F. Left posterior cerebellum (uvula of vermis). BA= Brodmann's area; S= superior face; I= inferior face, A= anterior face; P= posterior face; R= right face; L= left face (Figure modified from Mercadillo RE et al. 2014. *J Neurol Sci* 347(1-2):50-8.).

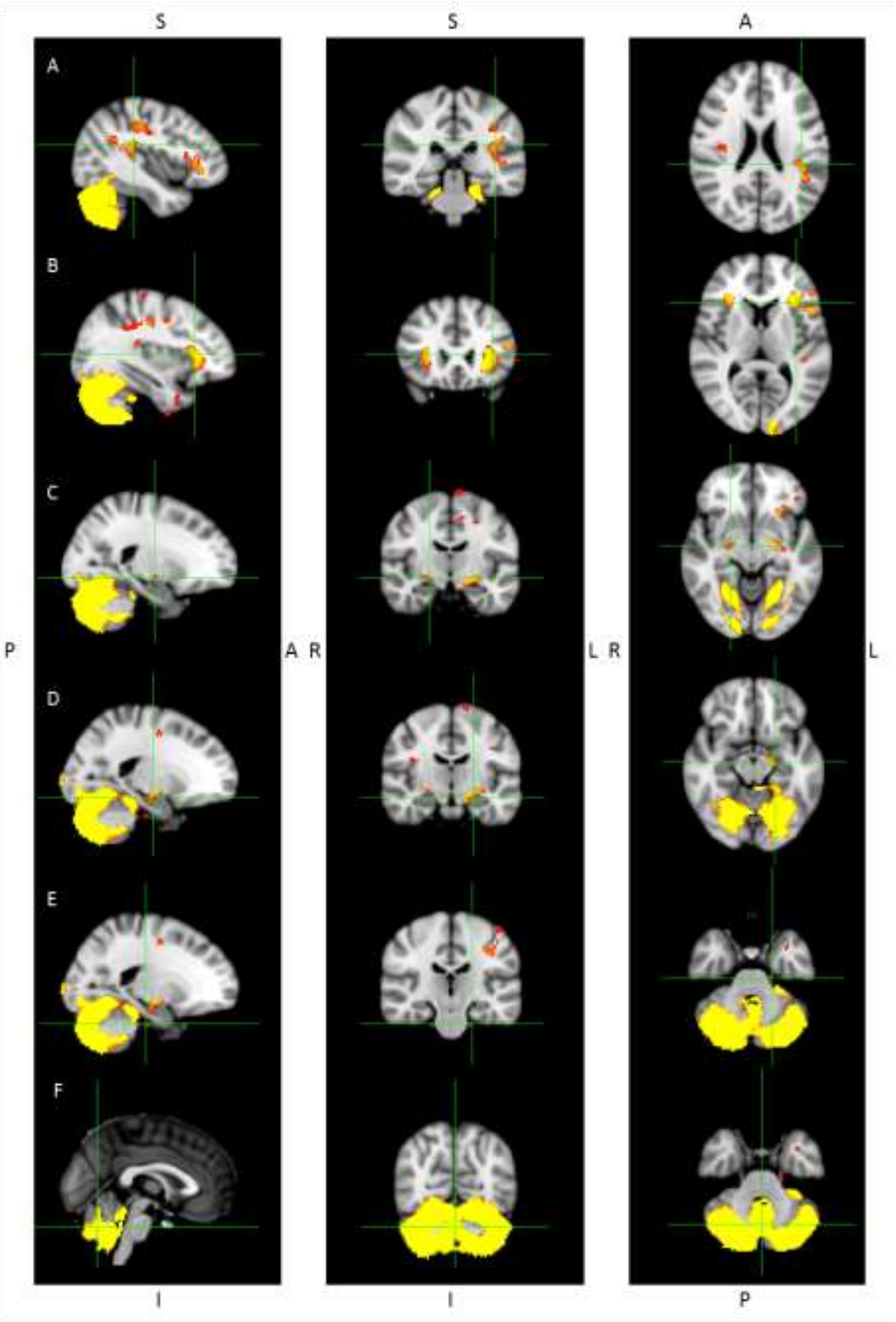

References

Andersson, J.L.R., Jenkinson, M., Smith, S., 2007. Non-linear registration, aka Spatiatial normalisation: FMRIB technical report TR07JA2.

Good, C. D., Johnsrude, I. S., Ashburner, J., Henson, R. N., Friston, K. J., Frackowiak, R. S., 2001. A voxel-based morphometric study of ageing in 465 normal adult human brains. Neuroimage. 14(1 Pt 1), 21-36. doi:10.1006/nimg.2001.0786.

Mercadillo RE, Galvez V, Diaz R, Hernandez-Castillo CR, Campos-Romo A, Boll MC, et al. Parahippocampal gray matter alterations in Spinocerebellar Ataxia Type 2 identified by voxel based morphometry. *J Neurol Sci* (2014) **347**(1-2):50-8. doi: 10.1016/j.jns.2014.09.018

Smith, S. M., Jenkinson, M., Woolrich, M. W., Beckmann, C. F., Behrens, T. E., Johansen-Berg, H., Bannister, P.R., De Luca, M., Drobnjak, I., Flitney, D.E., Niazy, R.K., Saunders, J., Vickers, J., Zhang, Y., De Stefano, N., Brady, J.M., Matthews, P.M., 2004. Advances in functional and structural MR image analysis and implementation as FSL. Neuroimage. 23(Suppl 1), S208-219. doi: 10.1016/j.neuroimage.2004.07.051.
